# DOPING CONTROL IN MALE SOCCER PLAYERS IN BRAZIL: 10 YEARS OF FOLLOW-UP

**DOI:** 10.1590/1413-785220243201e273282

**Published:** 2024-03-22

**Authors:** Herman Fabian Moscovici, Paulo Henrique Schmidt Lara, Fernando Antonio Gaya Solera, Moisés Cohen, Jorge Roberto Pagura, Gustavo Gonçalves Arliani

**Affiliations:** 1 Centro de Traumatologia do Esporte da Escola Paulista de Medicina, São Paulo, SP, Brazil.; 2 Pesquisador independente; 3 Faculdade de Medicina do ABC, Santo André, SP, Brazil.

**Keywords:** Doping in Sports, Soccerz, Athlete, Professional, Epidemiology, Doping nos Esportes, Futebol, Atletas Profissionais, Epidemiologia

## Abstract

**Objective:**

To understand the Adverse Analytical Finding (AAF) that have occurred in Brazilian soccer in a recent 10-year period, comparing them to international data, to know the Brazilian profile.

**Methods:**

A review of the AAR in the Doping Control Commission database of the Brazilian Football Association from 2008 to 2017. The AAR in professional male soccer players between 2008 and 2017 were considered.

**Results:**

The sample selected in this research was composed of 40,092 doping tests, with 113 AAR, identified in 18 different competitions (0.28%) in the professional category, in Brazilian national and state competitions between 2008 and 2017, flagged in doping control exams through urine samples. Stimulants were detected most frequently (31.0%), followed by glucocorticoids (21.2%), diuretics, and masking agents (19.5%). The Brazilian Championship series did not show a relationship with any of the World Anti-Doping Agency (WADA) groups of substances. Series A showed 0.07% of AAR, Series B 0.21%, Series C 0.75% and Series D 1.49.

**Conclusion:**

The rate of AAR in Brazilian soccer was 0.28%, lower than the average for all soccer worldwide, and shows similar percentages among field positions. Stimulants were the most prevalent drugs. The national elite soccer competitions showed significantly fewer cases than the lower divisions. ***Level of Evidence II; Retrospective Study.***

## RESUMO

**Objetivo:**

Compreender os Resultados Analíticos Adversos (RAA) ocorridos no futebol brasileiro nos últimos 10 anos, comparando-os aos dados internacionais, para conhecer o perfil do futebol brasileiro.

**Métodos:**

Revisão dos RAA no banco de dados da Comissão de Controle de Doping da Confederação Brasileira de Futebol de 2008 a 2017. Foram consideradas os RAA entre 2008 e 2017.

**Resultados:**

A amostra selecionada nesta pesquisa foi composta por 40.092 exames antidoping com 113 RAA, os quais foram identificados em 18 competições diferentes (0,28%) em atletas da categoria professional, entre 2008 e 2017, sinalizadas em exames de controle de doping através de amostras de urina. Estimulantes foram detectados com maior frequencia (31%), seguidos de glicocorticoides (21,2%), diuréticos e agentes mascarantes (19,5%). A série do Campeonato Brasileiro não apresentou relação com nenhum dos grupos de substâncias da World Anti-Doping Agency (WADA). A série A apresentou 0,07% da AAR, Série B 0,21%, Série C 0,75% e Série D 1,49%.

**Conclusão:**

A taxa de RAA no futebol brasileiro foi de 0,28%, inferior à media do futebol mundial e apresenta percentuais semelhantes entre as posições do campo. Os estimulantes foram as drogas mais prevalentes. As competições nacionais de futebol das Séries superiores apresentaram significativamente menos casos do que as inferiores. ***Nível de Evidência II; Estudo Retrospectivo***.

## INTRODUCTION

Soccer is the most popular sport globally, with the most significant number of players. Its professional league is coordinated nationally by its confederations, which report to the continental confederations that, in turn, report to FIFA (*Fédération Internationale de Football Association*). Concerned about ethical aspects, physical and mental health, and equality among competitors, FIFA has been attentive to the doping problem in the sport since 1966. In 1970, regular anti-doping control activities began for international soccer matches and competitions.^1^

In 1999, the International Olympic Committee (IOC) founded the World Anti-Doping Agency (WADA) to organize, coordinate, and promote an international fight against doping, independently and institutionally. Today WADA produces the content, methods, and guidelines that coordinate all anti-doping actions in the main sports played worldwide.^2^

In Brazil, doping control in soccer is organized and managed by the Commission for Doping Control (CCD) of the Brazilian Football Brazilian Football Association (CBF), in partnership with Brazilian Doping Control Authority (ABCD).

The data published so far in soccer present only the percentage of adverse analytical results (AAF) by the total samples collected per year and the most prevalent group of drugs. According to this information, the most common drugs in world soccer are anabolic agents (S.1) and Stimulants (S.6), the same characteristic observed in general data of all sports, published annually by WADA^3^. European soccer follows these statistics, with the group of anabolic agents (S.1) as the most prevalent.^4^ In Brazil, the most common drugs are not known, as well as the annual percentages of AAF, so more detailed information about the athlete’s profile involved in doping cases is necessary.

The proposal is to understand the AAF in a recent 10-year period, comparing with international data, identifying the most detected agents, the prevalent age, the field position, the division in which the athlete was playing, trying to relate these variables to know the Brazilian profile.

## MATERIAL AND METHODS

The study was approved by institutional board under the number 0750/2019. A review of AAFs from 2008 to 2017 was conducted in the CBF CCD database. Data were accessed through an encrypted, exclusive access program, preserving the athlete’s anonymity. The information contained: athlete’s age, club, competition played at the time of the test, identified substance group, and type of punishment in months. The study considered the AAF that occurred in professional male players in Brazilian soccer between 2008 and 2017. Inclusion criteria: participants in national soccer competitions (A, B, C, D series and Brazil Cup) and state championships, totaling 20 championships. Exclusion criteria: female soccer athletes, athletes in youth category championships, and international competitions. The variables considered in the study were: athlete’ position -goalkeeper, defender, midfielder, striker -, type of league - national, series A, B, C, D and Brazil Cup, state championships -, class of substance found - from S1 to S9, according to WADA’s official list, as shown in Table 1, type of punishment - less than six months, six to 12 months, 12 to 18 months, more than 18 months, acquitted -, demographic analysis - considering the minimum and maximum age, mean, median, standard deviation.

**Table 1:** General characteristics of flagged athletes.

Age (years) (n=113)	Mean	27.2	
	Median	28.0	
	Minimum-maximum	18-41	
	Standard Deviation	4.7	
Age group (n=113)	Up to 23 years old	27	23.9%
	24 to 30 years old	63	55.8%
	31 years old or more	23	20.4%
Year (n=113)	2008	10	8.8%
	2009	9	8.0%
	2010	10	8.8%
	2011	8	7.1%
	2012	7	6.2%
	2013	12	10.6%
	2014	6	5.3%
	2015	11	9.7%
	2016	19	16.8%
	2017	21	18.6%
Position (n=113)	Goalkeeper	16	14.2%
	Defender	41	36.3%
	Midfielder	31	27.4%
	Striker	25	22.1%
Competition (n=113)	Baiano	1	0.9%
	Brazilian A Series	11	9.7%
	Brazilian B Series	24	21.2%
	Brazilian C Series	6	5.3%
	Brazilian D Series	4	3.5%
	Carioca	6	5.3%
	Cearense	2	1.8%
	Gaúcho	8	7.1%
	Goiano	1	0.9%
	Mineiro	4	3.5%
	Paulista A1	9	8.0%
	Paulista A2	8	7.1%
	Paulista A3	9	8.0%
	Paulista Second Division	2	1.8%
	Pernambucano	5	4.4%
	Brazil Cup	10	8.8%
	Northeast Cup	1	0.9%
	Out of competition	2	1.8%
Championship (n=113)	National	55	48.7%
	State	58	51.3%
Punishment (n=113)	Up to six months	48	42.5%
	6 to 12 months	21	18.6%
	12 to 18 months	1	0.9%
	More than 18 months	25	22.1%
	Acquitted	15	13.3%
	No information	3	2.7%

The inferential analyses, used to confirm or refute evidence found in the descriptive analysis, were:

- Student-t-test for independent samples;^5^ Analysis of Variance (ANOVA) with a fixed factor,^6^ and Mann-Whitney^7^ comparing age, according to the use of a forbidden substance, athlete’s position in the game, type of competition, and time of punishment.
- Pearson’s chi-square and Fisher’s exact test^8^ (or its extension) to study the association between the use of a forbidden substance and the athlete’s position in the game, type of competition, and time of punishment.

In all the conclusions obtained through the inferential analyses, the alpha significance level of 5% was used.

The statistical analysis was done through the mean, median, minimum and maximum values, standard deviation, absolute and relative frequencies (percentage).

The statistical analyses were performed using the program IBMSPSS Statistics, version 24.^9^

## RESULTS

The sample selected in this research was composed of 113 AAF in male soccer athletes, professional category, in Brazilian national and state competitions between 2008 and 2017, caught in doping control exams through urine samples. The average age of these athletes was 27.2 years, ranging from 18 to 41 years; a bit more than half were between 24 and 30 years old (55.8%). Considering the athlete’s position in the game, about 41 (36.3%) were defenders, 31 (27.4%) midfielders, 25 (22.1%) strikers, and 16 (14.2%) goalkeepers. Approximately half of the athletes were identified during national competitions (48.7%) and had corresponding punishments, mostly up to six months (42.5%). The stimulants group was most frequently detected (31.0%), followed by glucocorticoids (21.2%), diuretics and masking agents (19.5%), anabolic agents (15.0%), cannabinoids (8.0%), growth factors or peptide hormones (4.4%), metabolic modulators or hormones (3.5%), and, finally, beta-2 agonists (0.9%). It is important to note that no athletes tested positive for narcotics. In this research, the relation of age, playing position, competitions grouping the national and state tournaments, the type of championship, and punishment were important objects of investigation, according to doping control tests on the athletes.

Athletes who used substances of the class of metabolic modulators or hormones had higher age when compared to those who did not use them (p=0.008). The athletes’ age was not the same, according to the type of competition (p=0.025); athletes from the D series presented a higher age when compared to the A and C series of the Brazilian Championship. Age showed no relationship with the other information described in Table 2.

**Table 2:** Summary measures of athletes’ age (years), according to anti-doping use, position, competition, championship and punishment.

	mean	median	minimum	maximum	Standard deviation	p
**Anabolic agents**						
Have not used (n=96)	27.3	28.0	18.0	41.0	4.8	0.578^a^
Used (n=17)	26.6	26.0	21.0	34.0	3.8	
**Growth factors, peptide hormones**						
Have not used (n=108)	27.2	28.0	18.0	41.0	4.7	0.955^b^
Used (n=5)	27.6	26.0	26.0	34.0	3.6	
Beta-2 agonist						
Have not used (n=112)	27.3	28.0	18.0	41.0	4.7	0.325^b^
Used (n=1)	23.0	23.0	23.0	23.0		
**Metabolic modulators or hormones**						
Have not used (n=109)	27.0	27.0	18.0	41.0	4.6	0.008^a^
Used (n=4)	33.3	33.0	30.0	37.0	3.8	
**Diuretics or masking agents**						
Have not used (n=91)	26.9	27.0	18.0	39.0	4.5	0.171^a^
Used (n=22)	28.5	28.0	19.0	41.0	5.4	
**Stimulants**						
Have not used (n=78)	27.2	27.0	19.0	41.0	4.5	0.460^b^
Used (n=35)	27.4	28.0	18.0	35.0	5.1	
**Narcotics**						
Have not used (n=113)	27.2	28.0	18.0	41.0	4.7	-
Used	-	-	-	-	-	
**Cannabinoids**						
Have not used (n=104)	27.2	27.5	18.0	41.0	4.7	0.887^a^
Used (n=9)	27.4	29.0	22.0	32.0	3.8	
**Glucocorticoids**						
Have not used (n=89)	27.5	28.0	18.0	41.0	4.7	0.248^a^
Used (n=24)	26.3	26.0	19.0	39.0	4.5	
Total (n=113)	27.2	28.0	18.0	41.0	4.7	
**Position**						
Goalkeeper (n=16)	25.4	25.0	21.0	33.0	3.9	0.146^c^
Defense (n=41)	27.9	28.0	19.0	35.0	4.0	
Midfield (n=31)	28.1	28.0	18.0	41.0	5.8	
Striker (n=25)	26.2	27.0	18.0	34.0	4.4	
**Brazilian Competition**						
Series A (n=11)	25.5	26.0	18.0	34.0	4.7	0.025^c^
Series B (n=24)	27.3	28.0	19.0	34.0	4.0	
Series C (n=6)	24.8	23.0	21.0	32.0	4.3	
Series D (n=4)	32.5	33.0	29.0	35.0	2.6	
**Championship**						
National (n=55)	27.6	28.0	18.0	39.0	4.6	0.392^a^
State (n=58)	26.9	26.0	18.0	41.0	4.8	
**Punishment**						
0 - 6 months (n=48)	26.7	27.0	18.0	34.0	3.9	0.884^c^
6 - 12 months (n=21)	27.3	27.0	18.0	41.0	5.8	
12 - 18 months (n=1)	28.0	28.0	28.0	28.0	-	
> 18 months (n=25)	27.4	28.0	19.0	37.0	5.0	
Acquitted (n=15)	28.1	29.0	19.0	36.0	5.1	

a Student-t-test for independent samples,

b Mann-Whitney,

c Analysis of Variance (ANOVA) with a fixed factor.

**Figure 1:**
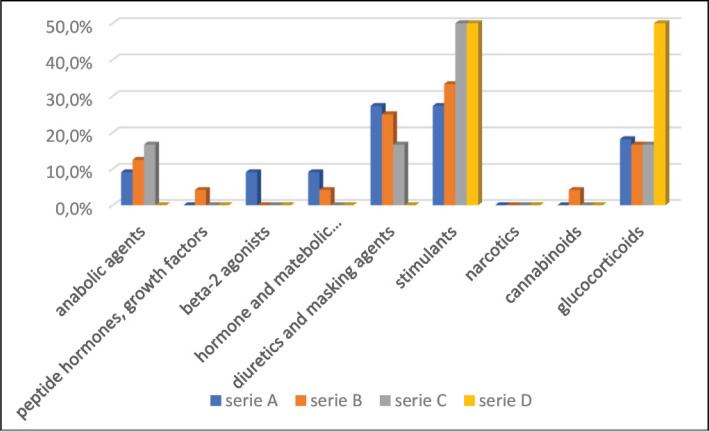
Distribution of the athletes in the Brazilian Championship Series, according to category of WADA banned substance used.

**Figure 2:**
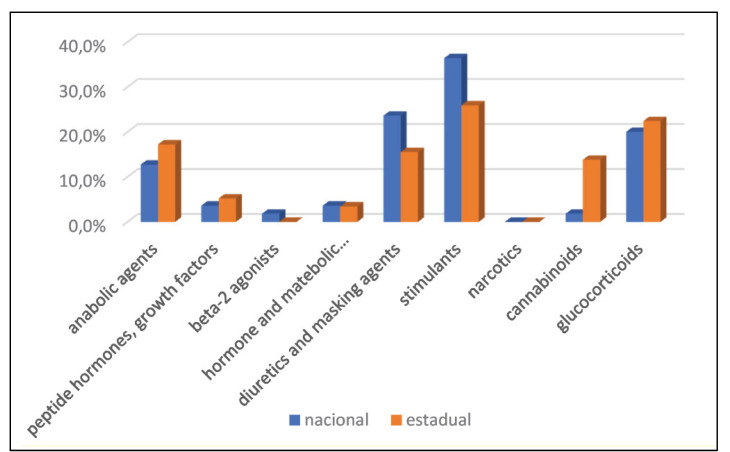
Distribution of the type of championship, according to anti-doping results.

The position in the game was related only to the use of three groups of substances: growth factors or peptide hormones (p=0.026) among strikers; diuretics or masking agents (p=0.042) among goalkeepers and midfielders, and glucocorticoids (p=0.043) among goalkeepers and defenders (Table 3). The Brazilian Championship series has not shown a relationship with any WADA substance groups. Higher cannabinoid use was confirmed among athletes during state championships compared to national ones. The other relationships were not significant. The distribution of the type of championship, according to substance results, can be seen in Figure 1 and the distribution of the type of championship, according to anti-doping results can be seen in Figure 2.

**Table 3:** Distribution of the athletes’ field position, according to anti-doping use.

	Position								
	Goalkeeper		Defender		Midfielder		Striker		p	
**Anabolic agents**									
Have not used	13	81.3%	34	82.9%	26	83.9%	23	92.0%	0.733^d^
Used	3	18.8%	7	17.1%	5	16.1%	2	8.0%	
**Growth factors, peptide hormones**									
Have not used	16	100.0%	40	97.6%	31	100.0%	21	84.0%	0.026^d^
Used	-	-	1	2.4%	-	-	4	16.0%	
Beta-2 agonist									
Have not used	16	100.0%	41	100.0%	30	96.8%	25	100.0%	0.637^d^
Used	-	-	-	-	1	3.2%	-	-	
**Metabolic modulators or hormones**									
Have not used	16	100.0%	41	100.0%	28	90.3%	24	96.0%	0.137^d^
Used	-	-	-	-	3	9.7%	1	4.0%	
**Diuretics or masking agents**									
Have not used	11	68.8%	36	87.8%	21	67.7%	23	92.0%	0.042^d^
Used	5	31.3%	5	12.2%	10	32.3%	2	8.0%	
**Stimulants**									
Have not used	13	81.3%	30	73.2%	21	67.7%	14	56.0%	0.327^e^
Used	3	18.8%	11	26.8%	10	32.3%	11	44.0%	
**Narcotics**									
Have not used	16	100.0%	41	100.0%	31	100.0%	25	100.0%	-
Used	-	-	-	-	-	-	-	-	
**Cannabinoids**									
Have not used	15	93.8%	36	87.8%	31	100.0%	22	88.0%	0.159^d^
Used	1	6.3%	5	12.2%	-	-	3	12.0%	
**Glucocorticoids**									
Have not used	12	75.0%	27	65.9%	27	87.1%	23	92.0%	0.043^e^
Used	4	25.0%	14	34.1%	4	12.9%	2	8.0%	

d Fisher’s exact test extension,

e Pearson’s chi-square.

Athletes with more than 18 months of punishment used anabolic agents more often, than the other ones (p=0.008). The time of punishment did not show a significant relationship with the other substance groups. (Table 4)

**Table 4:** Distribution of athletes’ punishment period, according to anti-doping results.

	Punishment									
	Up to 6 months		6 to 12 months		12 to 18 months		More than 18 months		Acquitted		p
	N	%	N	%	N	%	N	%	N	%	
**Anabolic agents**											
Have not used	45	93.8	20	95.2	1	100.0	16	64.0	13	86.7	0.008^d^
Used	3	6.3%	1	4.8	-	-	9	36.0	2	13.3	
**Growth factors, peptide hormones**											
Have not used	44	91.7	21	100.0	1	100.0	25	100.0	15	100.0	0.281^d^
Used	4	8.3	-	-	-	-	-	-	-	-	
**Beta-2 agonist**											
Have not used	47	97.9	21	100.0	1	100.0	25	100.0	15	100.0	>0.999^d^
Used	1	2.1	-	-	-	-	-	-	-	-	
**Metabolic modulators or hormones**											
Have not used	47	97.9	21	100.0	1	100.0	24	96.0	13	86.7	0.221^d^
Used	1	2.1	-	-	-	-	1	4.0	2	13.3	
**Diuretics or masking agents**											
Have not used	38	79.2	17	81.0	-	23	92.0	10	66.7	0.109^d^
Used	10	20.8	4	19.0	1	100.0	2	8.0	5	33.3	
**Stimulants**											
Have not used	36	75.0	14	66.7	1	100.0	14	56.0	11	73.3	0.498^d^
Used	12	25.0	7	33.3	-	-	11	44.0	4	26.7	
**Narcotics**											
Have not used	48	100.0	21	100.0	1	100.0	25	100.0	15	100.0	-
Used	-	-	-	-	-	-	-	-	-	-	
**Cannabinoids**											
Have not used	41	85.4	20	95.2	1	100.0	25	100.0	15	100.0	0.150^d^
Used	7	14.6	1	4.8	-	-	-	-	-	-	
**Glucocorticoids**											
Have not used	38	79.2	13	61.9	1	100.0	23	92.0	11	73.3	0.132^d^
Used	10	20.8	8	38.1	-	-	2	8.0	4	26.7	

There is no relationship between the athlete’s position in the game and punishment duration (p=0.831).

Between 2008 and 2017, the lowest rate of AAF occurred in 2014 (0.14%), and the highest rate occurred in 2017 (0.42%). A total of 30,498 samples were collected at national competitions and 9,444 samples at state competitions. Among the AAF, we observed almost half of the cases in national competitions and the other half in state competitions.

Comparing proportionally, the AAF rate obtained was 0.57% in state championships and 0.18% in national championships. In the national competitions, samples collected from the A, B, C, D series and the Brazil Cup were included in the analysis.

It was observed that the A Series presented 0.07% of AAF, followed by B Series (0.21%), C Series (0.75%), D Series (1.49), and Brazil Cup (0.34%).

Within the state competitions, the Paulista Championship, in its various divisions, showed the highest incidence of AAF, with the A3 Series being the most prevalent, followed by the Paulista B Series and the Northeast Cup.

## DISCUSSION

Between 2008 and 2017, 40,092 urine samples were collected, with 113 cases of AAF indicating substances banned in athletes, in or out of competition, by the WADA anti-doping list. There are no studies that compare the number or percentage of AAF between countries in soccer. Al Ghobain et al.^10^ analyzed Saudi Arabia’s total AAF, including all sports in the country, and noted an average of 3.1% over nine years.

Similarly, Kioukia-Fougia et al.^11^ did a similar study in Greece and found an average of 1.42% over seven years. These data may have several biases because they add sports with very different characteristics. Aguilar-Navarro separated team and individual sports in his research; among the individual sports, he obtained an AAF of 1.6% (+/- 0.9%), and among team sports, an AAF of 1.7% (+/- 0.6%), based on worldwide data from WADA.^10^

With an average AAF of 0.28% over ten years, Brazilian soccer is well below the average found in statistical surveys that include several sports. Starting in 2008, Brazilian soccer has always had lower AAF percentages than the sum of soccer results from the rest of the world, according to data published by WADA. This may be related to the extensive testing work carried out in Brazil and the strict punishments for athletes and professionals involved in flagged and judged cases. Still comparing Brazilian soccer to the world soccer concerning the types of substances most commonly found, we noticed that, in the data provided by WADA from 2014, when the publications specifying the groups of drugs by sports began, it was possible to stratify, within soccer, which substances were the most common.^3^ In contrast to our research, Brazilian soccer obtained the presence of stimulants (S6) as the most common group of substances, followed by glucocorticoids (S9) and diuretics or masking agents (S5). It was also observed that diuretics represent double the incidence in Brazilian soccer, compared to data from soccer worldwide, according to WADA.^3^

### Age

The average age found in the study was 27.2 years. A greater concentration at the extremes of the ages was expected, due to immaturity among younger athletes or the search for performance among older athletes. In the 4th division of Brazilian soccer, a greater presence of positive tests amongst athletes over 30 years old was observed, possibly related to the end of their career, corroborating the hypothesis of an alternative search for performance.

Similarly, the substances most commonly found in athletes over 30 years old are metabolic modulators or hormones (S4). Thevis et al. correlate this class of drugs to the treatment of sarcopenia, loss of muscle mass, and bone mass, which could attract older athletes seeking high performance.^11^ We believed that younger athletes would be more affected by social drugs, such as stimulants (S6) and cannabinoids (S8), which was contradicted by the study.

### Substance

In Brazilian soccer, the class of drugs most identified in the study was stimulants, more specifically group 6A, in which cocaine is found, with 56% of the total AAF, demonstrating a problem of social order, which interferes directly in the practice of soccer. Cocaine has the ability to stimulate adrenergic neurotransmitters and can generate performance improvement. However, its use is more related to social problems than searching for better sports performance.^12^ By discussing the presence of drugs of abuse as a cause of AAF, other actors identified Tetrahydrocannabidiol (THC) as the main drug.^13^ Kiouki-Fougia et al,^11^ when studying the prevalence of substances in doping tests in Greece between 2005 and 2011, found THC in second place among total substances, representing 10% of cases, losing to anabolic agents, which accounted for 31%. Strano Rossi et al.^14^ noted the highest prevalence of THC and secondarily cocaine among drugs of abuse in his study, which included 100,000 urine tests of young athletes in Italy over ten years.^11^ All these studies demonstrate the real gravity of Brazilian soccer concerning the abuse of cocaine, the main drug found in the tests done in Brazil.

An important factor is that there were no flagrant cases of substance use in the narcotics class in this 10-year period.

### Position

The statistically significant presence between drug classes by position is not clear.

Hormone peptides and growth factors (S2) were observed, statistically related to strikers, diuretics or masking agents (S5) among midfielders and goalkeepers, and glucocorticoids (S9) among goalkeepers and defenders. There is no relationship in the literature between position played on the field and the demand for a particular class of drugs. Attention is drawn to the proportion of positive cases among goalkeepers, due to a smaller number of athletes per team, in this group of analysis, in comparison with the other groups of positions in the field.

### Punishment

There are four main reasons for an athlete to be acquitted after having an AAF in a doping test: negative counterevidence for identified substance, the existence of Therapeutic Authorization (TRA) for the use of the caught substance, proof of administration of the drug without the athlete’s knowledge, and contamination or error in sample handling.

In our analysis, the most common type of punishment includes a period of absence, around six months, enough time to generate a financial loss to the club, physical and sportive loss to the athlete, as well as social inconveniences with the public disclosure of a “positive” case.

Anabolic agents (S1) were responsible for the longest time away from the sport, around 18 months. Doping in sport shows how complex it is to combat. Geographic and cultural differences are fundamental to an understanding and better control of these cases, as well as the professionals involved, who support the athletes. Pielke, in an editorial, discusses how demographic and social factors should be considered to understand the risk factors in athletes involved with doping.^15^

In their study, Morente-Sánchez et al.^16^ applied a questionnaire to 237 soccer professionals in Spain and found that 57.6% did not know what WADA meant and 84.9% did not know the list of banned substances. According to Hon et al.^17^, the low percentage rate of AAF surveilled by WADA annually is underestimated. Their study, researching the “real number” of doping through interviews with athletes, estimates a number around 14% to 39%. Ulrich et al.^18^, with a similar survey, estimated a figure around 43.6%. Until now, no statistical survey on doping control has been seen that analyzes the variables of a single sport discipline, as described in this article in detail, which can serve as a first step to investigate triggering factors and an epidemiological profile. This article analyzes athletes with substantial social, geographical, and financial distinctions, presenting biases in the comparison between tournaments with low annual testing and tournaments testing in all rounds, which may increase the percentage value, even in a few cases.

### Limitations

The presence of 113 AAFs is a small number for definitive statistical correlations, especially taking into account the various analysis variables used in this study. We understand that many alerts were given with this information, and that the continuity in the collection of this information and the constant analysis could represent more significant results.

## CONCLUSION

In the analysis between 2008 and 2017, the rate of AAF in Brazilian soccer is 0.28%, lower than the summed average of all soccer worldwide, and shows similar percentages among the positions on the field. The average age is around 27 years old. Stimulants are the most prevalent drugs, followed by glucocorticoids and diuretics and masking agents. The elite national soccer competitions have far fewer cases compared to the lower divisions.
